# Genetic variant rs2243115 of the IL-12/IL-35 pathway contributes to the risk of coronary artery disease

**DOI:** 10.7150/ijms.102562

**Published:** 2025-04-13

**Authors:** Qianwen Chen, Wenjuan Zhang, Tian Xie, Jiangtao Dong, Junxia Zhang, Lingfeng Zha

**Affiliations:** 1 Department of Pediatric Cardiology, Maternal and Child Health Hospital of Hubei Province, Tongji Medical College, Huazhong University of Science and Technology, Wuhan 430070, China.; 2 Department of Cardiology, Union Hospital, Tongji Medical College, Huazhong University of Science and Technology; Hubei Key Laboratory of Biological Targeted Therapy, Union Hospital, Tongji Medical College, Huazhong University of Science and Technology; Hubei Provincial Engineering Research Center of Immunological Diagnosis and Therapy for Cardiovascular Diseases, Union Hospital, Tongji Medical College, Huazhong University of Science and Technology, Wuhan 430022, China.; 3 Department of Geriatrics, The Central Hospital of Wuhan, Tongji Medical College, Huazhong University of Science and Technology, Wuhan 430022, China.; 4 Department of Cardiovascular Surgery, Union Hospital, Tongji Medical College, Huazhong University of Science and Technology, Wuhan 430022, China.; 5 Department of Cardiology, Nanjing First Hospital, Nanjing Medical University, Nanjing 210006, China.

**Keywords:** Coronary artery disease, rs2243115, IL12A, IL-12, IL-35

## Abstract

**Background:** Coronary artery disease (CAD) involves inflammation. IL-12p35, a common subunit of both IL-12 and IL-35, is encoded by the *IL12A* gene and is a potential therapeutic target in CAD. We probed into the genetic relationships between *IL12A* and CAD in a Chinese Han population to provide a novel potential target and a theoretical basis for the anti-inflammatory therapies in CAD.

**Materials and Methods:** In total, 768 patients with CADs and 768 controls were recruited for a case-control association analysis of the functional genetic variant rs2243115 of *IL12A*. Allelic and genotypic associations between rs2243115 and CAD and its subgroup were assessed by Logistic regression analysis. Additionally, multiple linear regression analysis was performed to explore the association between rs2243115, serum lipid levels and CAD severity. Bioinformatic tools were used to predict the potential function of rs2243115.

**Results:** Our results showed no differences in the allele and genotype frequency distribution of rs2243115 between patients with CAD and controls. The subgroup analysis found no association between rs2243115 and CAD in either male or female groups. Furthermore, rs2243115 was not related to early- or late-onset CAD, or CAD severity. However, we did observe that rs2243115 was negatively related to HDL-c level (*P*=0.016,* β***=-**0.063) and positively related to LDL-c level (*P*=0.029, *β*=0.058). Biological function prediction indicated many functional elements in the rs2243115 region, suggesting that rs2243115 may regulate gene expression in the IL-12/IL-35 pathway.

**Conclusion:** The functional genetic variant, rs2243115, of *IL12A*, may play a role in CAD by regulating the IL-12/IL-35 pathway and affecting lipid levels and inflammatory responses, thereby providing a potential therapeutic target for CAD.

## Introduction

Coronary artery disease (CAD) is an atherosclerotic disease. When the stenosis of the coronary artery lumen exceeds 50%, coronary artery circulation disorder occurs, resulting in an insufficient myocardial blood supply, angina pectoris, and other clinical symptoms. When the lumen of the coronary artery is completely blocked, myocardial ischemia, hypoxia, and necrosis result in myocardial infarction (MI). CAD occurs more often in individuals over 40 years of age and more often in men than in women; more than 7 million people worldwide die from CAD every year, causing a huge social and economic burden^1^, ^2^. Many factors contribute to the development of CAD, including environmental factors (lifestyle factors, body mass index [BMI], gender, age, etc.) and genetic factors, of which genetic factors are estimated to account for approximately 40-60%^3^, ^4^. Several CAD susceptibility genes have been identified using various means, such as genome-wide association study (GWAS), which involve various inflammatory factors^5^. Existing evidence indicates that inflammation plays a key role in all stages of atherosclerosis and involves various immune cells^6^-^8^. Our previous study showed that inflammatory factors secreted by different immune cells, such as IL-13^9^, IL-9^10^, IL-23^11^, and TSLP^12^, are associated with CAD susceptibility.

IL-12 and IL-35 are highly homologous heterodimeric factors belonging to the IL-12 family of cytokine. IL-12 promotes cellular immune response by activating the STAT4 signal transduction pathway to produce IFN-γ and can produce pro-inflammatory effects in the pathological process of CAD via modulating Th1 cell response. A previous study found increased plasma IL-12 concentrations in patients with CAD, and a significant increase in plasma IL-12 levels was found in patient with unstable angina and stable angina, especially in the unstable angina group; the plasma hs-CRP level was positively correlated with IL-12^13^. Moreover, high expression of IL-12 mRNA and IL-12 protein was detected in human atherosclerotic plaques, and IL-12 upregulated the Th1 cell response^14^. IL-35 is an immunosuppressive factor involved in regulating inflammation by enhancing the inhibitory activity of Tregs^15^. Studies have reported that IL-35 may play a protective role in CAD. Recent research has found that IL-35 levels of the stable CAD group were considerably lower than the control group^16^ and the administration of IL-35 suppresses the progression of atherosclerosis in Apoe^-/-^ mice^17^. However, IL-35 plasma concentrations increase in patients with CAD^18^. IL-12p35 is a common subunit of both IL-12 and IL-35, is encoded by *IL12A*, and may play a pivotal role in the biological processes of IL-12 and IL-35. Recent research has found that the lack of IL-12p35 improves cardiac repair after MI by facilitating angiogenesis^19^. This indicates that IL-12p35 is a potential therapeutic target for CAD.

**Figure 1:**
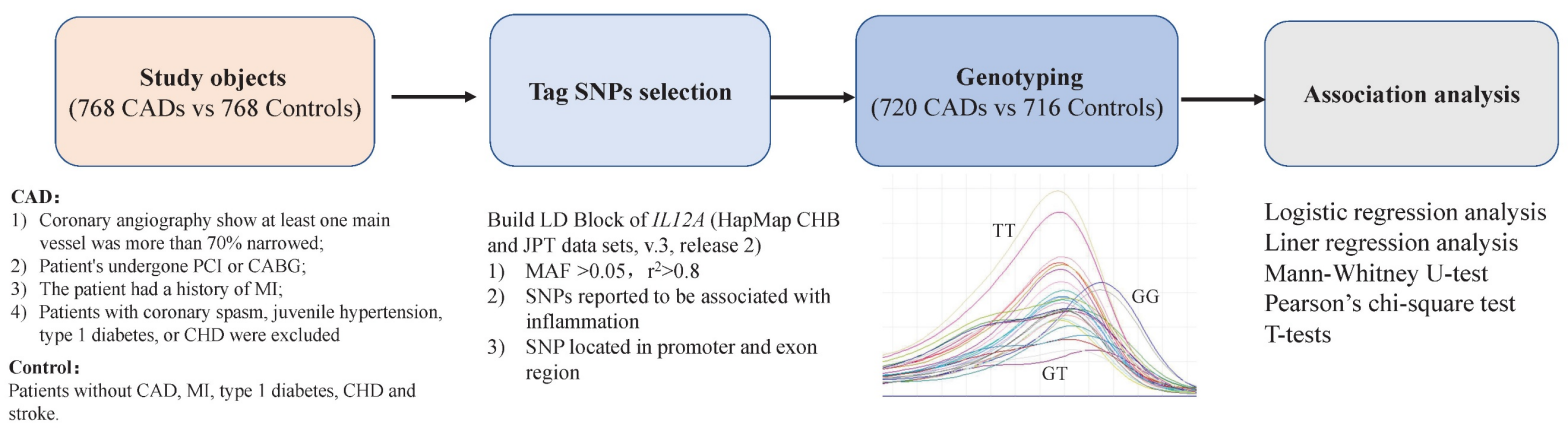
Framework for the study.

Currently, anti-inflammatory therapy is a focal point in the field of cardiovascular research, and it is crucial to identify suitable and effective anti-inflammatory therapy targets^20^, ^21^. This case-control study aimed to preliminarily investigate the genetic connection between the *IL12A* gene and CAD to provide a potentially novel target and a theoretical foundation for anti-inflammatory therapy in CAD (Figure 1).

## Materials and Methods

### Study objects

The inclusion criteria for CAD were^9^, ^10^, ^12^: 1) coronary angiography showing major vessel narrowing >70%; 2) myocardial revascularization (percutaneous coronary intervention [PCI] or coronary artery bypass graft [CABG]); 3) patient has had MI; 4) coronary spasm, juvenile hypertension, type 1 diabetes, or congenital heart disease (CHD) were eliminated. Patients without CAD, MI, type I diabetes, CHD, or stroke were included as controls (Figure 1). The collected clinical information included age, gender, BMI, blood lipid levels, smoking status, history of diabetes and hypertension, and coronary angiography results, etc.

Our study included 768 patients with CADs and 768 controls who were recruited at the Union Hospital, Tongji Medical College, Huazhong University of Science and Technology (Wuhan, China) from January 2014 and November 2017. Participants' information was strictly confidential, and informed consent was obtained from all participants. Our study met the tenets of the World Medical Association Declaration of Helsinki and was approved by the Union Hospital, Tongji Medical College, Huazhong University of Science and Technology (0157-01). All the participants provided written informed consent.

### Tag single nucleotide polymorphism (SNP) selection

**Figure 2:**
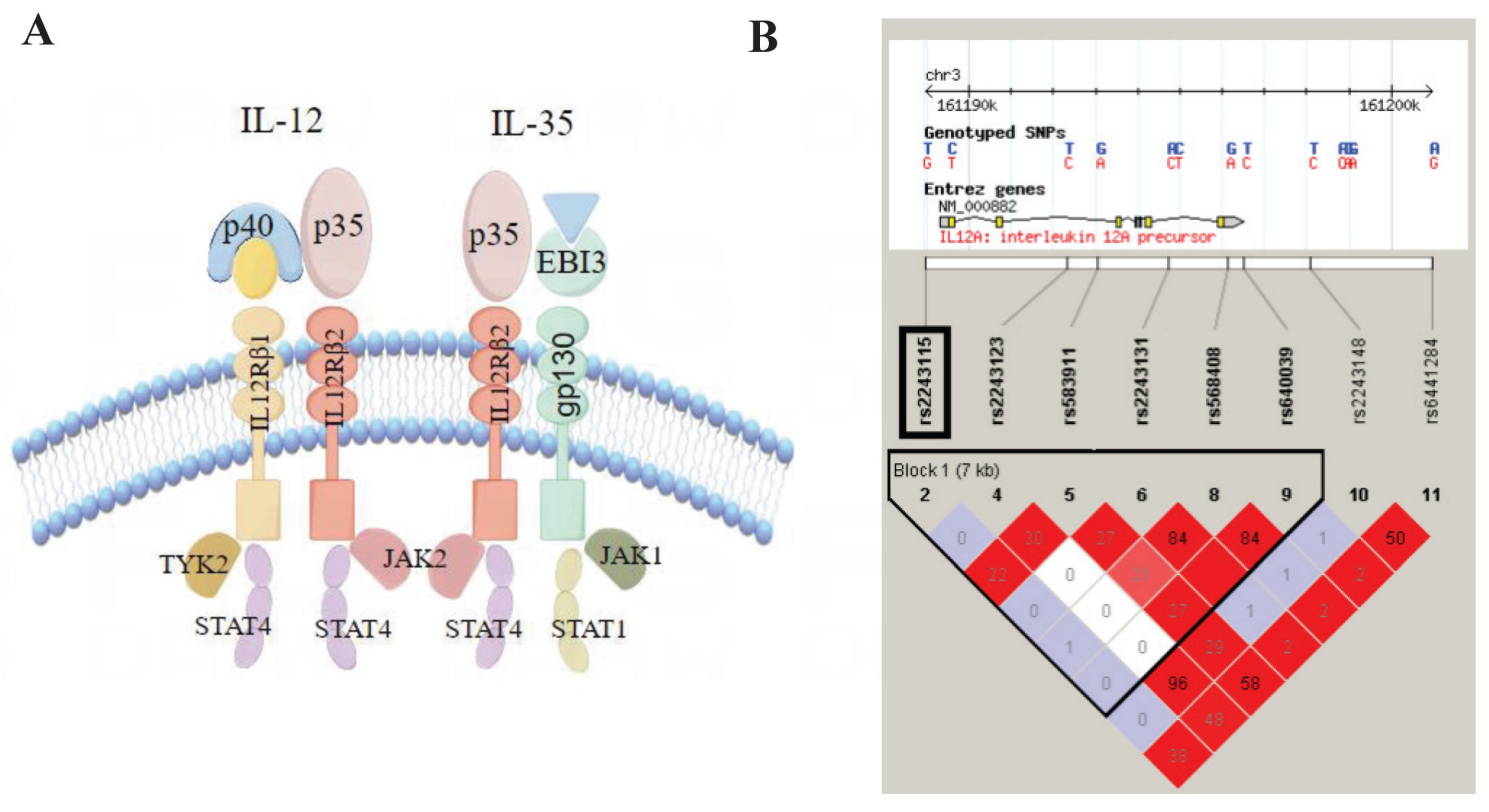
** Structure of IL-12/IL-35 pathway and Haploview linkage disequilibrium (LD) block map of *IL12A*.** A. Structure of IL-12/IL-35 pathway; B. LD block map of *IL12A*.

First, we built an LD Block map from HapMap (CHB and JPT datasets, V.3, release 2) covering *IL12A* and its upstream and downstream 5kbp region (Figure 2). Next, according to the following rules, we selected Tag SNPs^12^, ^22^: 1) minor allele frequency (MAF) >0.05, r^2^ >0.8, 2) SNPs reported to be related to inflammation, and 3) SNPs located in the regulatory or coding region (Figure 1). Finally, we chose rs2243115 as our research priority because rs2243115 is located in the promoter region of *IL12A* gene with an MAF of 0.1034, and has been reported to be related to inflammation.

### DNA acquisition and typing

DNA was extracted from blood using commercial kits (CW 2087). DNA amplification was carried out in the PCR reaction system (25μL) containing 2.5μL 10×PCR buffer, 0.5μL dNTP, 0.5μL forward primer (5'-AGCCAGCTCTCATCCCTTTT-3') and 0.5μL reverse primer (5'-ACACCCAGGGAGAACAGGA-3'), 1μL DNA, 0.5μL Taq DNA polymerase, 0.5μL SYTO 9 and 19μL double steaming water with TAKARA-TP600 PCR Amplifier (Takara, Japan) and genotyped using a Rotor-Gene 6000 High Resolution Melt (HRM) system (Cobette Life Science, Australia)^11^. Furthermore, we randomly selected 48 CADs and 48 controls to validate the HRM genotyping results by Sanger sequencing, using the forward primer of 5'-ACCTCCCTCAACCCTTCATG-3' and reverse primer of 5'-GACGTAGAGAGAGGAGTGCC-3'. The concordance rate for HRM genotyping was 100%. Genotyping was completed successfully in 93.75% of CAD cases and 93.23% of controls.

### Function prediction

The HaploReg database (http://archive.broadinstitute.org/mammals/haploreg/haploreg.php) integrates a large number of GWAS and expression Quantitative Trait Loci (eQTLs) results as well as epigenetic information such as histone ChIP-seq. This database contains information on SNP-linked sites in the genome and regulatory elements involved in various tissues or cell lines.

RegulomeDB (http://www.regulomedb.org/) annotates noncoding SNPs and gene regulatory elements, including eQTL, DNase I hypersensitive binding region (DNase), transcription factor binding site and known promoter region, etc. According to these notes, the website comprehensively scores SNPs, and the regulatory mechanisms in which SNPs of interested may participate can be predicted according to the scores, thus providing ideas for subsequent research.

The UCSC genome browser (http://genome.ucsc.edu/) contains information on gene regulation, including molecular transcription, DNA modification, histone modification, transcription factor binding, chromosome topology and so on. Based on the information from these databases, the possible regulatory mechanism of rs2243115 can be preliminarily understood.

### Statistical analysis

The Hardy-Weinberg equilibrium (HWE) test was performed using PLINK software (v.1.07). Allelic and genotypic analyses were carried out by chi-square contingence tables (2×2 and 2×3, respectively). Odds ratio (OR) and 95% confidence interval (CI) were assessed using SPSS (v.26.0). Allelic and genotypic associations were performed by logistic regression analysis and multiple logistic regression was conducted to correct for traditional risk factors of CAD. Multiple linear regression analysis explored the associations between rs2243115, serum lipid levels, and CAD severity (SPSS, v.26.0). *P*<0.05 indicates a statistically significant difference.

## Results

### Population characteristics

There were obvious differences in the clinical data between the CAD and control groups (all *P*<10^-3^). The CAD group had a higher mean age and BMI than the control group. The proportions of male and individuals with hypertension and diabetes were also higher in the CAD group than in the control group. Serum lipid levels of Tch, TG and LDL-c in patients with CAD were higher than those in the controls, but HDL-c levels were lower than those in the controls (Table 1).

### Associations between rs2243115 and CAD

Rs2243115 did not deviate from the HWE test in the control population (*P*=0.117). The MAF of the patients with CAD was 0.091 and that of the controls is 0.076. The allelic association analysis showed no association between rs2243115 and CAD before or after adjustment (*P*_obs_=0.131, *P*_adj_=0.666). In the genotypic association analysis, rs2243115 was related to CAD in additive mode (GG/GT/TT) and dominant mode (GG+GT/TT) (*P*_obs_=0.017 for additive mode and *P*_obs_=0.049 for dominant mode). However, when correcting for traditional risk factors, no association was observed between rs2243115 and CAD in the additive mode and dominant mode (*P*_adj_=0.662, OR, 1.080 [95% CI, 0.766-1.522] for additive mode, *P*_adj_=0.445, OR, 1.152 [95% CI, 0.802-1.655] for dominant mode, respectively). In the recessive model (GG/GT+TT), there was no association between rs2243115 and CAD either before or after adjustment (*P*_obs_=0.093, *P*_adj_=0.148) (Table 2).

### Subgroups analysis for the associations between rs2243115 and CAD

We divided the population into different groups, considering the effects of age and gender. Such as CAD was segmented into early-onset CAD and late-onset CAD based on the onset age of patients^10^, ^23^-^25^: early-onset CAD was in males ≤ 55 years old and females ≤ 65 years old, while late-onset CAD was in males > 55 years old and females > 65 years old.

In male group, rs2243115 was insignificant in the allelic and genotypic association analyses before or after adjusting risk factor (all *P*>0.05). While in female group, rs2243115 was related to CAD in the additive mode (GG/GT/TT) (*P*_obs_=0.047), but the association disappeared after risk factor correction (*P*_adj_=0.382, OR, 1.294 [95% CI, 0.726-2.307]) (Table 3).

Rs2243115 was not associated with late-onset CAD both in allelic or genotypic analyses (*P*_obs_ and *P*_adj_ >0.05). It was found that rs2243115 was related to early-onset CAD in the additive mode (GG/GT/TT) (*P*_obs_=0.044), but the association disappeared after risk factor correction (*P*_adj_=0.131, OR, 1.471 [95% CI, 0.892-2.426]) (Table 4).

### Associations between rs2243115 and severity of CAD

**Figure 3:**
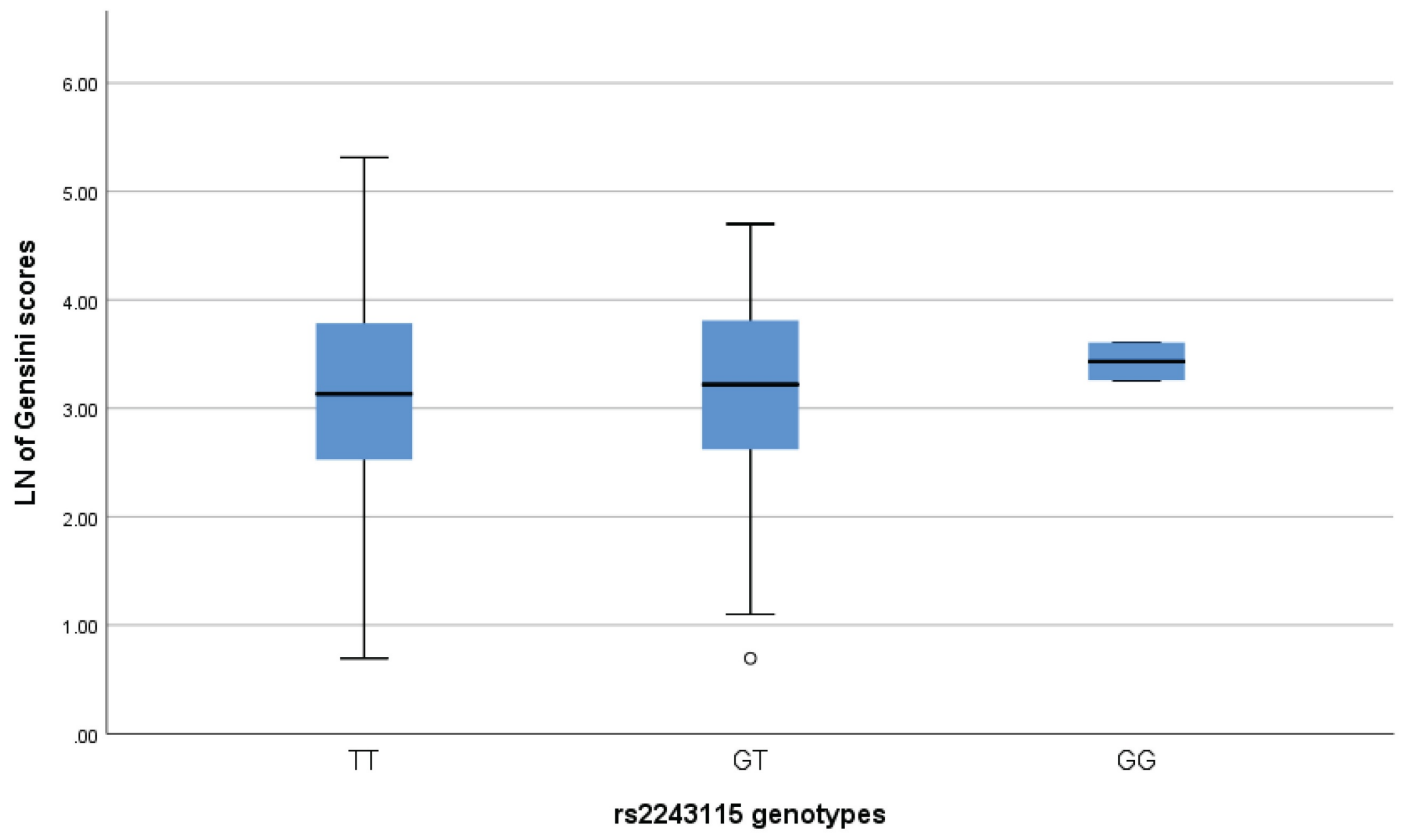
Association analysis between the LN of Gensini scores and the genotypes of rs2243115.

We used the Gensini score based on coronary angiography to assess the severity of CAD^26^, ^27^. The coronary segments were scored as 32, 16, 8, 4, 2 and 1 for 100%, 99-91%, 90-76%, 75-51%, 50-26% and 25-0%, respectively. The coronary segment's score is multiplied by a coefficient based on the vessel's importance and size (0.5-5.0) to obtain the Gensini scores. Gensini scores were obtained for 564 patients with CAD, but no difference was found in Gensini scores among carriers of the three genotypes for rs2243115 (*P*>0.05) (Figure 3). In addition, linear regression analysis and the Mann-Whitney U-test revealed no association between rs2243115 and CAD severity in either allele and genotype modes (*P*>0.05) (Table 5).

### Associations between rs2243115 and serum lipid levels

**Figure 4:**
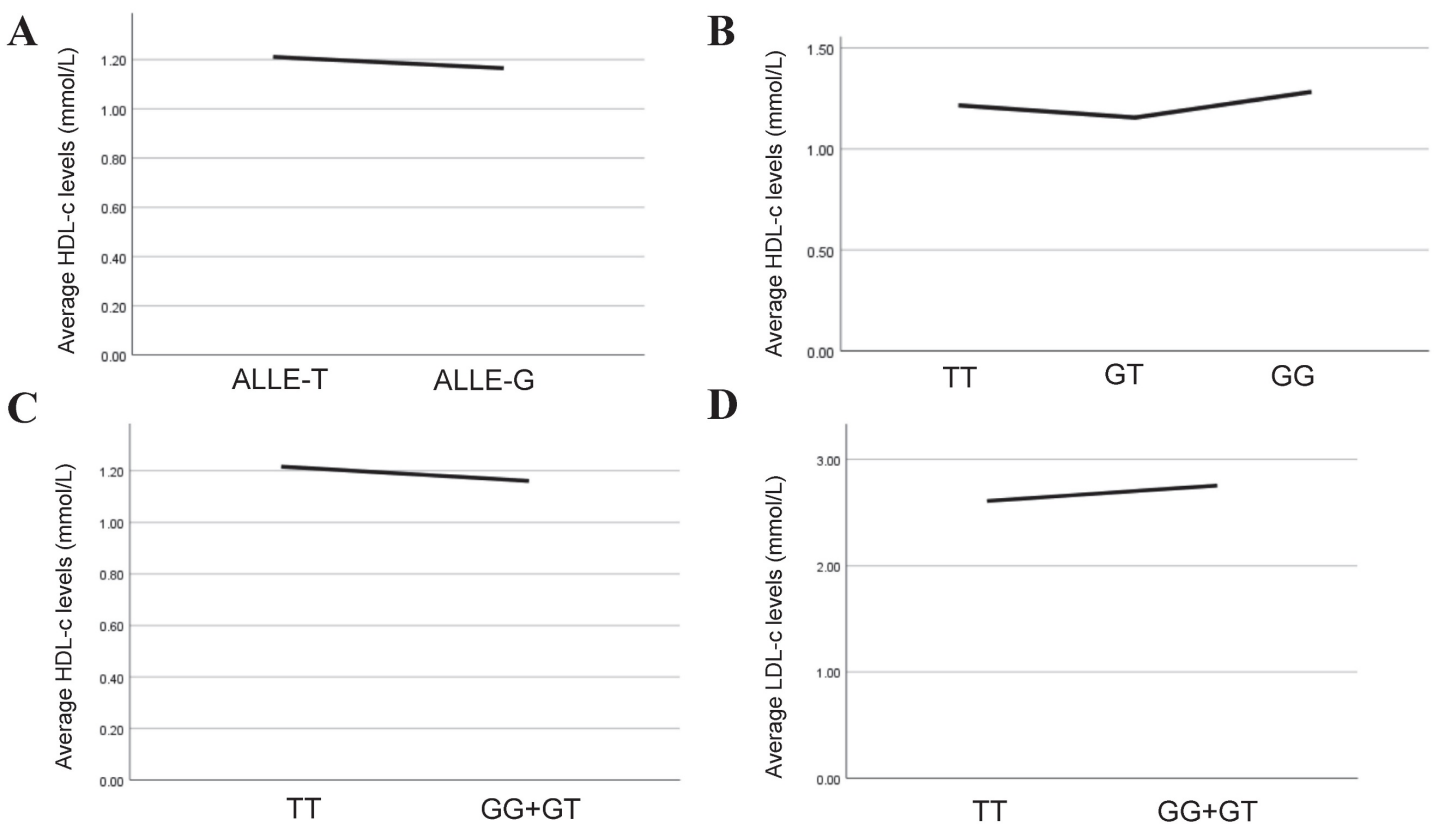
** Association analysis between serum lipid levels and the allelic and genotypes of rs2243115.** A. Association between HDL-c level and allelic of rs2243115; B. Association between HDL-c level and genotypes of rs2243115 for additive mode; C. Association between HDL-c level and genotypes of rs2243115 for dominant mode; D. Association between LDL-c level and genotypes of rs2243115 for dominant mode.

Lipids are independent risk factor of CAD and play a key role in its genesis and progression of CAD. In our study, multiple linear regression was implemented to explore further the association between rs2243115 and serum lipid levels in allelic and genotypic analyses. The result showed that in the allelic analysis, rs2243115 was significantly related to HDL-c (*P*=0.035,* β***=-**0.039). In genotypic analysis, rs2243115 was significantly associated with HDL-c (*P*=0.034,* β***=-**0.056 for additive mode and *P*=0.016,* β***=-**0.063 for dominant mode) (Table 6, Figure 4). In addition, it was found that rs2243115 was correlated with LDL-c in the dominant mode (*P*=0.029, *β***=**0.058) (Table 6, Figure 4).

### Potential function of rs2243115

**Figure 5:**
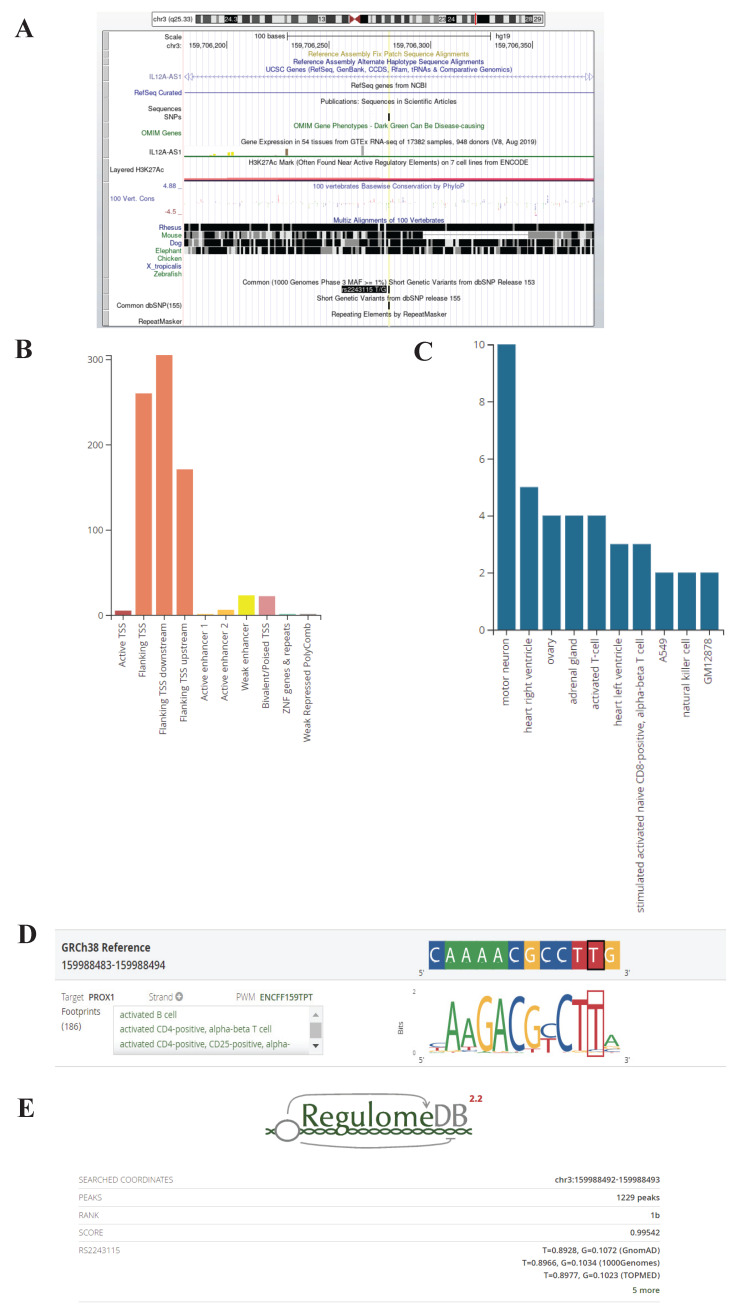
** Biological function prediction of rs2243115.** A. Rs2243115 prediction from UCSC Genome Browser; B, C, D. Rs2243115 prediction from RegulomeDB; E. Score of rs2243115 from RegulomeDB (https://www.regulomedb.org/regulome-search?regions=chr3%3A159988492-159988493&genome=GRCh38).

Rs2243115 is located upstream of the *IL12A* gene, the promoter region that may regulate gene expression. The UCSC database predicted that rs2243115 is located in a region containing various regulatory elements, such as the H3K27AC marker (Figure 5). The RegulomeDB database rated rs2243115 as 1b, which suggests that it has the eQTL+TF binding+any motif+DNase footprint+DNase peak maker and indicates that rs2243115 is a functional variant that is likely to regulate gene expression (Figure 5). The HaploReg database predicts that rs2243115 has a large number of promoter histone markers, enhancer histone markers, DNAse markers, and transcription factor binding sites in various tissues and cell lines, such as Blood & T-cell/B-cell and Heart &Aorta. In addition, rs2243115 is associated with target gene expression in numerous tissues, and multiple regulatory markers in loci are highly linked to rs2243115.

## Discussion

This case-control study investigated the association between the common genetic variant rs2243115 of *IL12A*, which encodes IL-12 and IL-35 shared subunits IL-12p35, and CAD in a Chinese Han population. Our results indicated no association between rs2243115 and CAD. Furthermore, subgroup analyses revealed no association between rs2243115 and CAD in either the male or female groups. Additionally, rs2243115 was not related to early- or late-onset CAD, or to CAD severity. However, rs2243115 negatively correlated with HDL-c levels and positively correlated with LDL-c levels.

The* IL12A* gene is located on chromosome 3. Although existing studies have suggested that the *IL12A* gene may be associated with CAD susceptibility, our study found that the genetic variant rs2243115 in* IL12A* was not associated with CAD. In a large-scale GWAS of CAD, we found no correlation between rs2243115 and CAD or MI (https://www.cardiogramplusc4d.org/). Similarly, rs2243115 were not statistically relation to the risk of CAD in the Chinese Zhuang population^28^. However, recent studies have found that in the southern region of Chinese Han population, the G allele of rs2243115 is independently correlated with an increased risk of CAD^29^. Our study was carried out in the central region of the Chinese Han population, and there may be regional differences. In addition, their study corrected for only the common risk factors of CAD, such as gender, age, BMI, smoking status and alcohol consumption, whereas our study corrected for more CAD risk factors, such as lipid levels. Lipid level is the most paramount risk factor for CAD; if not included in the correction, there may be bias in the results, and our follow-up study found that there is indeed a relationship between rs2243115 and lipid levels. The Genetics of Atherosclerotic Disease (GEA) Mexican study recently indicated that rs2243115 is related to a decreased risk of premature CAD^30^. However, our study found that rs2243115 was not associated with early- or late-onset CAD. We speculate that, on the one hand, there are population and racial differences between the two studies; on the other hand, the inclusion criteria of CAD in the two studies are different. CAD patients with >50% coronary stenosis on angiography was selected in their studies, whereas CAD patients with >70% coronary stenosis on angiography were selected in our study. In additional, a previous study only adjusted for CAD risk factors such as age, gender, BMI, and smoking. Furthermore, no relationship was found between rs2243115 and CAD severity. Recent research also found no association between rs2243115 genotypes and cognitive decline in CAD patients over 2 years^31^.

*IL12A* gene encode IL-12p35, a common subunit of IL-12 and IL-35 (Figure 2). Although IL-12 and IL-35 are IL-12 family members with similar structures, they have different pro-inflammatory or anti-inflammatory effects and functions, and both participate in the occurrence and development of atherosclerosis. IL-12 is a pro-inflammatory cytokine involved in Th1 differentiation; the production of IL-12 contributes to the progression of atherosclerotic plaque^32^. IL-35 is an anti-inflammatory cytokine mainly produced by Tregs, and IL-35 treatment reduces atherosclerotic plaque in Apoe^-/-^ mice^33^. Recent studies have shown that IL-35 reduces ox-LDL-induced atherosclerotic effects by regulating CAD-related miRNAs^34^. Thus, IL-12p35 may be pivotal in the IL-12/IL-35 pathway. Recent studies have shown that the use of IL-12p35 neutralizing antibodies prevented AMI-induced inflammatory cells from infiltrating the heart and ameliorated angiogenesis and heart function^19^. Lack of IL-12p35 aggravated the Th17/Treg imbalance and improved Apoe^-/-^ mouse atherosclerosis^35^. Through biological function prediction, the region rs2243115 was identified many promoter histone markers, enhancer histone markers, DNAse markers, and transcription factor binding sites in various tissues and cell lines, such as Blood & T-cell/B-cell and Heart &Aorta. Thus, rs2243115 may regulate IL-12p35 expression and participate in CAD.

Our results indicated no direct association between rs2243115 and CAD, but we found rs2243115 was negatively related to HDL-c levels (*P*=0.016,* β***=-**0.063) and positively related to LDL-c levels (*P*=0.029, *β*=0.058). Hyperlipidemia is known to be the main risk factor for CAD, among which an increase in LDL-c is the main cause of the occurrence and development of atherosclerosis. In contrast, HDL-c is a protective factor for atherosclerosis. A clinical observational study found that treating patients with heterozygous familial hypercholesterolaemia with atorvastatin decreased the abundance of IL-12p35 mRNA in mononuclear cells^36^. Furthermore, one study showed that increased serum IL-12 levels were correlated with increased hs-CRP and LDL levels^37^. This evidence indicates that the IL-12/IL-35 pathway is not only involved in inflammation and immunity but is also related to lipid metabolism. Complex interactions between lipid levels and inflammation lead to the formation and progression of atherosclerotic plaques^38^. When a variety of risk factors cause vascular endothelial dysfunction, LDL enters and persists under the endoderm, activates the endothelium after modification, induces a large number of inflammatory cells, especially monocytes, to enter the local area, phagocytes lipids to form foam cells and forms a cascade of amplified inflammatory response, leading to the formation and progression of atherosclerotic plaques. With persistence inflammation, plaque instability results in plaque rupture, fissures or erosion, and thrombosis, resulting in myocardial damage and necrosis. Lowering LDL-c can significantly reduce the incidence of cardiovascular events for CAD patients. Therefore, lipid-lowering therapy is the primary treatment for CAD^39^, ^40^. Recent studies have found that blocking cytokine pathways, such as the IL‑1β/IL‑6 pathway to control residual inflammation risk, can significantly reduce cardiovascular events for CAD patients under active use of lipid-lowering drugs^41^. Experts believe that a combination of lipid-lowering and anti-inflammatory therapy will be the best treatment for CAD^42^, ^43^. However, regulation of the inflammatory response is extremely complex, and identifying key and effective inflammatory regulatory targets is key to the success of anti-inflammatory therapy. According to our results, we speculate that rs2243115 may regulate the expression of IL-12p35 and then affect the IL-12/IL-35 pathway, further regulating lipid metabolism and the inflammatory response and thus participating in the process of atherosclerosis. The IL-12/IL-35 pathway, which plays a dual role in regulating lipid metabolism and the inflammatory response, is expected to be a promising target for CAD therapy. Further in-depth mechanistic research and clinical trials are required to explore its roles in the future.

This study has several limitations. Firstly, the study was conducted exclusively within the central Chinese Han population, which may have led to variations among different races and regions. Secondly, the sample size was limited, necessitating further expansion for future verification. Thirdly, there is a lack of laboratory data concerning the inflammatory state of patients and the expression levels of IL-12 and IL-35. Finally, although we predicted the potential function of rs2243115, further detailed mechanistic studies are required to elucidate how rs2243115 exerts its regulatory role.

## Conclusion

We found that the functional genetic variant rs2243115 of *IL12A*, which encodes IL-12p35, a common subunit of IL-12 and IL-35, might play roles in CAD by regulating the IL-12/IL-35 pathway and affecting lipid levels and the inflammatory response, which provides a potential therapeutic target for CAD.

## Figures and Tables

**Table 1:** The characteristics of the study population.

Characteristics	CAD (n=720)	Control (n=716)	*P*
Age (years)	63.45±10.99	61.20±10.47	<10^-3^
Male (%)	70.69	50.42	<10^-3^
Smoking (%)	46.25	27.79	<10^-3^
BMI (kg/m^2^)	24.25±1.55	23.48±1.41	<10^-3^
Hypertension (%)	68.75	52.09	<10^-3^
DM (%)	34.31	15.36	<10^-3^
Tch (mmol/L)	5.10±1.19	4.23±0.94	<10^-3^
TG (mmol/L)	1.80±1.17	1.36±0.88	<10^-3^
HDL-c (mmol/L)	1.12±0.30	1.30±0.32	<10^-3^
LDL-c (mmol/L)	2.98±1.02	2.28±0.70	<10^-3^

The data are shown as the mean ±SD. Categorical data were tested by chi-square tests, while measurement data were tested by *t*-tests. CAD, coronary artery disease; DM, diabetes mellitus; BMI, body mass index; Tch, total cholesterol; TG, triglyceride; HDL-c, high-density lipoprotein cholesterol; LDL-c, low-density lipoprotein cholesterol.

**Table 2:** Allelic and genotypic association analysis of rs2243115 with CAD.

Model	N (CAD)	N (Control)	*P*_obs_	*P*_adj_	OR (95%CI)
ALLE	131/1309	108/1324	0.131	0.666	1.078 (0.767-1.514)
DOM	129/591	101/615	0.049	0.445	1.152 (0.802-1.655)
REC	2/718	7/709	0.093	0.148	0.182 (0.018-1.834)
ADD	2/127/591	7/94/615	0.017	0.662	1.080 (0.766-1.522)

*P*_obs_, observed *P* value; *P*_adj_, *P* value adjusted by the covariates; OR, odds ratio after adjustment; ADD, additive mode, rs2243115_GG/GT/TT; DOM, dominant model, rs2243115_GG+GT/TT; REC, recessive model, rs2243115_GG/GT+TT.

**Table 3:** Allelic and genotypic association analysis of rs2243115 with CAD in gender subgroups.

Model	Male (CAD 509/Control 361)	Male (CAD 509/Control 361)	Male (CAD 509/Control 361)	Female (CAD 211/Control 355)	Female (CAD 211/Control 355)	Female (CAD 211/Control 355)
	*P*_obs_	*P*_adj_	OR (95%CI)	*P*_obs_	*P*_adj_	OR (95%CI)
ALLE	0.619	0.841	0.959 (0.634-1.449)	0.141	0.369	1.313 (0.725-2.377)
DOM	0.507	0.936	0.982 (0.634-1.522)	0.058	0.186	1.540 (0.812-2.920)
REC	0.375	0.315	0.109 (0.001-8.217)	0.294	0.239	0.183 (0.011-3.087)
ADD	0.496	0.835	0.956 (0.622-1.467)	0.047	0.382	1.294 (0.726-2.307)

*P*_obs_, observed *P* value; *P*_adj_, *P* value adjusted by the covariates; OR, odds ratio after adjustment; ADD, additive mode, rs2243115_GG/GT/TT; DOM, dominant model, rs2243115_GG+GT/TT; REC, recessive model, rs2243115_GG/GT+TT.

**Table 4:** Allelic and genotypic association analysis of rs2243115 with CAD in age subgroups.

Model	CAD-early-onset (CAD 225/ Control 716)	CAD-early-onset (CAD 225/ Control 716)	CAD-early-onset (CAD 225/ Control 716)	CAD-late-onset (CAD 495/Control 716)	CAD-late-onset (CAD 495/Control 716)	CAD-late-onset (CAD 495/Control 716)
	*P*_obs_	*P*_adj_	OR (95%CI)	*P*_obs_	*P*_adj_	OR (95%CI)
ALLE	0.221	0.135	1.464 (0.888-2.414)	0.200	0.501	0.866 (0.569-1.317)
DOM	0.096	0.077	1.625 (0.950-2.780)	0.101	0.604	0.889 (0.569-1.388)
REC	0.137	0.999	0.000 (0.000-0.000)	0.253	0.382	0.327 (0.027-4.005)
ADD	0.044	0.131	1.471 (0.892-2.426)	0.084	0.506	0.866 (0.567-1.323)

*P*_obs_, observed *P* value; *P*_adj_, *P* value adjusted by the covariates; OR, odds ratio after adjustment; ADD, additive mode, rs2243115_GG/GT/TT; DOM, dominant model, rs2243115_GG+GT/TT; REC, recessive model, rs2243115_GG/GT+TT.

**Table 5:** Allelic and genotypic association analysis of rs2243115 with severity of CAD.

Model	β	SE	*P*_adj_	*P*_mwu_
**ALLE**	0.003	0.011	0.913	0.761
**DOM**	0.002	0.020	0.957	0.810
**REC**	0.003	0.017	0.688	0.594
**ADD**	0.005	0.021	0.910	0.855

Association analysis between the LN of Gensini scores and the genotypes of rs2243115. The distribution difference of the LN of the Gensini scores in different genotypes of rs2243115 was compared by Mann-Whitney U-test. *P*_obs_, observed *P* value; *P*_adj_, *P* value adjusted by the covariates; OR, odds ratio after adjustment; ADD, additive mode, rs2243115_GG/GT/TT; DOM, dominant model, rs2243115_GG+GT/TT; REC, recessive model, rs2243115_GG/GT+TT.

**Table 6:** Allelic and genotypic association between rs2243115 and serum lipid levels.

Model	Serum lipid	β	SE	*P*
**ALLE**	Tch	0.004	0.027	0.147
	TG	-0.005	0.005	0.797
	HDL-c	-0.039	0.016	0.035
	LDL-c	0.036	0.006	0.055
**DOM**	Tch	0.040	0.008	0.128
	TG	0.001	0.009	0.964
	HDL-c	-0.063	0.030	0.016
	LDL-c	0.058	0.010	0.029
**REC**	Tch	0.003	0.002	0.921
	TG	-0.039	0.002	0.138
	HDL-c	0.019	0.006	0.478
	LDL-c	-0.017	0.002	0.530
**ADD**	Tch	0.038	0.009	0.146
	TG	-0.007	0.010	0.796
	HDL-c	-0.056	0.032	0.034
	LDL-c	0.051	0.011	0.054

Tch, total cholesterol; TG, triglyceride; HDL-c, high-density lipoprotein cholesterol; LDL-c, low-density lipoprotein cholesterol; ADD, additive mode, rs2243115_GG/GT/TT; DOM, dominant model, rs2243115_GG+GT/TT; REC, recessive model, rs2243115_GG/GT+TT.
